# Peptide Self-Assembled Nanocarriers for Cancer Drug Delivery

**DOI:** 10.1021/acs.jpcb.2c06751

**Published:** 2023-02-22

**Authors:** Vijay Bhooshan Kumar, Busra Ozguney, Anastasia Vlachou, Yu Chen, Ehud Gazit, Phanourios Tamamis

**Affiliations:** † The Shmunis School of Biomedicine and Cancer Research, George S. Wise Faculty of Life Sciences, Tel Aviv University, Tel Aviv 6997801, Israel; ‡ Artie McFerrin Department of Chemical Engineering, Texas A&M University, College Station, Texas 77843-3122, United States; § Department of Materials Science and Engineering, Iby and Aladar Fleischman Faculty of Engineering, Tel Aviv University, Tel Aviv 6997801, Israel; ∥ Department of Materials Science and Engineering, Texas A&M University, College Station, Texas 77843-3003, United States; ⊥ Sagol School of Neuroscience, Tel Aviv University, Tel Aviv 6997801, Israel

## Abstract

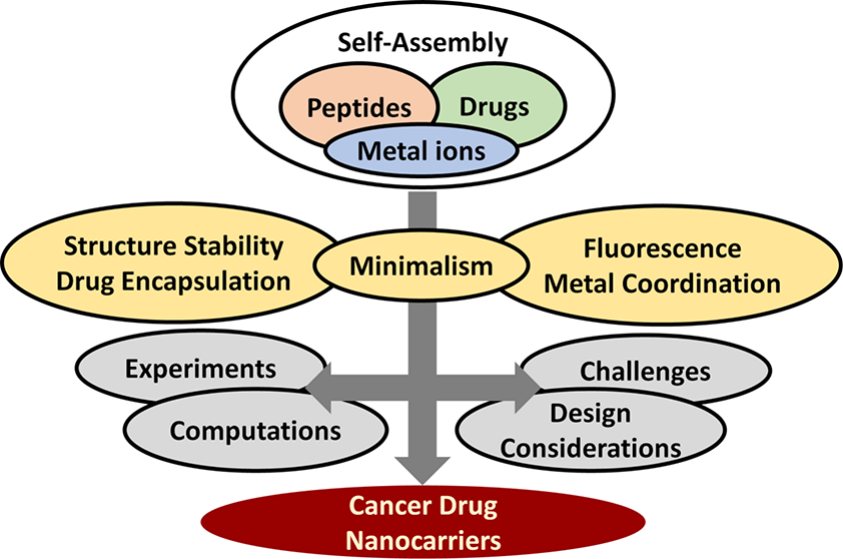

The design of novel cancer drug nanocarriers is critical in the framework of cancer therapeutics. Nanomaterials are gaining increased interest as cancer drug delivery systems. Self-assembling peptides constitute an emerging novel class of highly attractive nanomaterials with highly promising applications in drug delivery, as they can be used to facilitate drug release and/or stability while reducing side effects. Here, we provide a perspective on peptide self-assembled nanocarriers for cancer drug delivery and highlight the aspects of metal coordination, structure stabilization, and cyclization, as well as minimalism. We review particular challenges in nanomedicine design criteria and, finally, provide future perspectives on addressing a portion of the challenges via self-assembling peptide systems. We consider that the intrinsic advantages of such systems, along with the increasing progress in computational and experimental approaches for their study and design, could possibly lead to novel classes of single or multicomponent systems incorporating such materials for cancer drug delivery.

## Introduction

Both less and more economically developed countries suffer from cancer as a key cause of death,^1^ and cancer is considered the second leading cause of death, after cardiovascular diseases.^1^ There has therefore been a concerted effort to develop novel cancer therapeutics, leading to the approval of new cancer drugs by the FDA;^2^ interestingly, from 2000 to 2017, cancer therapy generated more accelerated, fast track, and priority approvals in comparison to other therapeutic areas.^3−6^ Cancer could be regarded as a plurality of different diseases rather than one disease; thus, in general, cancer and its treatment could be seen as a multifaceted problem. Treatments include surgery, radiotherapy, and chemotherapy, with the latter being the most commonly used for systemic treatment to suppress cancer cell proliferation, as well as disease progression and metastasis.^7^ Despite the fact that conventional chemotherapy has been partly successful, there are several challenging aspects associated with its application, such as low therapeutic indices, poor bioavailability, requirements of high doses, development of drug resistance, and nonspecific targeting, as well as adverse side effects.^8^

There are multiple problems to be addressed in chemotherapy which include, among others, the choice of drug(s) (i.e., active pharmaceutical ingredient(s)), the selection of the administration route for single and/or combinations of drugs, and the drug delivery system to allow optimal access to the target cancer cells.^2^ There is a variety of active pharmaceutical ingredients used for cancer treatments, such as small compounds, monoclonal antibodies, and peptides, as well as proteins. Small compounds still comprise the leading class for cancer therapeutic treatments, while antibodies are also becoming increasingly important.^2^ Cancer therapeutics can be administered in monotherapy or as combination regimens. As far as different routes of administration are concerned, these therapeutics are administered primarily orally, intravenously, and subcutaneously, with the last method having limited application than the former two.^2^

Importantly, drugs not only act against cancer cells but also can harm normal cells; adverse effects thus constitute a key challenge.^7^ Thus, drug delivery systems appear as a highly promising solution toward providing effective, noninvasive approaches toward the delivery of a particular drug or drug combinations at the right location and period of time and, additionally, minimize the effects on normal cells.^2^ In this context, nanomaterials are gaining increased interest as drug delivery systems for cancer drugs. Particularly, nanocarriers may combine a variety of advantageous properties, including but not limited to protecting drugs dissolved in the bloodstream, augmenting drug pharmacological and pharmacokinetic properties; enabling drug targeting to particular tissues and cells, thereby improving efficacy and limiting drug accumulations to organs such as kidney, liver, and spleen; delivering one or a combination of imaging and therapeutic molecules, which can also allow real-time monitoring.^7,9,10^

During the past decades, a series of nanomaterials have been investigated for their capacity to serve as cancer drug delivery systems, including but not limited to liposomes, polymers, dendrimers, micellar nanoparticles, and inorganic nanomaterials. While each category of nanomaterials has unique strengths, they also face key limitations: (a) Liposomes can be disadvantageous with respect to their distribution and removal mechanism or breakage in vivo; (b) polymers can be disadvantageous with respect to their inflammatory response and degradation pathway; (c) dendrimers can de disadvantageous due to immunoreaction and hematological toxicity; (d) micellar nanoparticles can be disadvantageous due to scale-up production and cytotoxicity; (e) inorganic materials can be disadvantageous due to metal toxicity, stability, and storage (reviewed in ref (7)).

Self-assembling peptides constitute an emerging novel class of highly attractive nanomaterials in biomedicine, with several applications including tissue regeneration and drug delivery; in drug delivery, they can be used to facilitate drug release and/or stability, as well as reduce side effects.^11^ Due to their diverse physicochemical properties, peptides can form diverse nanostructures with advantageous properties to conventional nonbiological materials.^12^ Self-assembling peptide nanostructures include but are not limited to nanoparticles, nanotubes, nanofibers, and hydrogels and have been widely studied for drug delivery applications.^13^ Particularly for cancer treatment, it is critical to consider the design of drug delivery systems capable of circumventing the different physiological, extracellular, and intracellular barriers. Self-assembling peptide materials have received significant interest because of their potentially tunable pharmacokinetic profile and drug targeting specificity.^14^

Self-assembling peptides’ potential biocompatibility, tunable bioactivity, and ability to be designed to (i) efficiently target particular sites, to load a variety of drugs and high load of drugs, as well as to (ii) possess triggered drug release at disease sites make them highly attractive candidates for drug delivery systems, including cancer drug delivery systems (reviewed in refs (11−13)). Also, while peptides may also be considered as drugs themselves,^12^ this Perspective focuses on self-assembling peptide materials which serve as drug delivery systems, specifically associated with cancer drugs.

## Peptide Self-Assembly and Formation of Peptide Nanostructures

**Figure 1:**
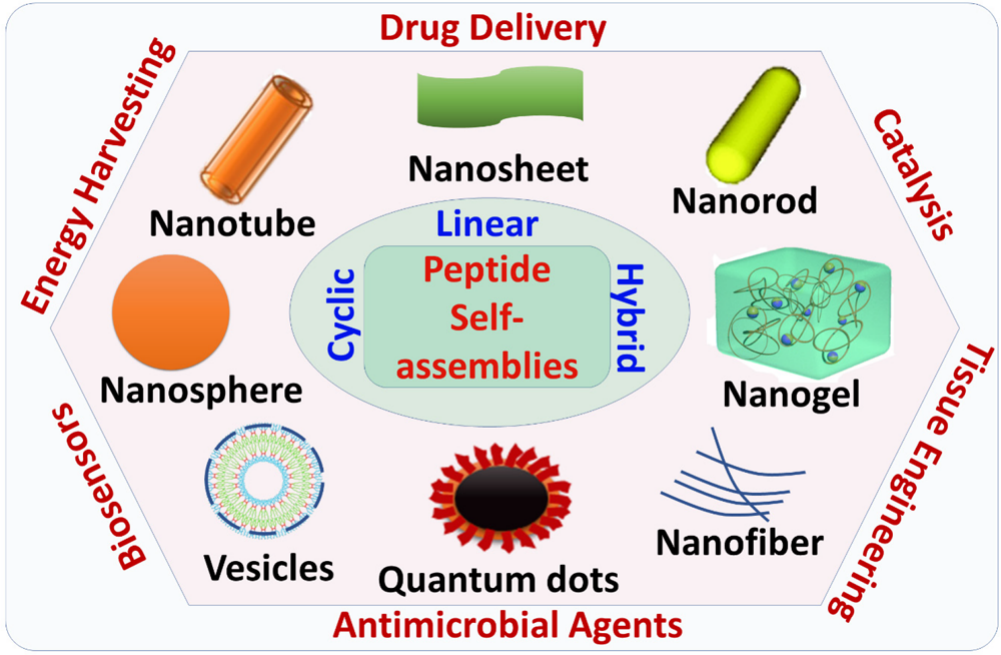
Overview of example cases of peptide self-assembled nanostructures and areas that they have been explored for their promising applications.

Self-assembling peptide systems can maintain structural integrity and stability via noncovalent interactions such as hydrophobic, π–π stacking, electrostatic, and hydrogen bonding.^15^ The factors that affect peptide self-assembly can be categorized into intrinsic and external.^16^ Intrinsic factors are associated with the type of peptide, including its length and its amino acid sequence, as well as the physicochemical properties of amino acid side chains.^12^ Examples of external factors affecting peptide self-assembly include pH, temperature, solvent, pressure, etc.^17^ Peptides, including linear, cyclic, and hybrid, can self-assemble into a wide variety of nanostructures, such as nanotubes, nanosheets, nanorods, nanogels, nanofibers, quantum dots, nanospheres, etc. (Figure 1), while peptide materials have been explored for a series of promising applications, including but not limited to several areas such as energy harvesting, catalysis, sensors, antimicrobial as well as tissue engineering agents, and drug delivery.^18−20^ Peptides can play a key role in drug delivery owing to their inherent advantages and their capacity to self-assemble into nanostructures. Given the developments in the fields of biotechnology, nanotechnology, and materials chemistry, many peptides have recently been explored for their capacity to serve as nanomaterials to deliver drugs (reviewed in refs (13, 19, and 21)). In this context, self-assembled peptide nanostructures can be advantageous with respect to their stability to enzymatic degradation biocompatibility, hydrophobic drug encapsulation, and sustained drug release; they can also possess advantageous shear-thinning viscoelastic and/or adjuvanting properties; in addition, they can serve as intracellular transporters as well as respond to physiological environment alterations.^22^ In this Perspective, we focus on peptide self-assembled nanocarriers, particularly for cancer drug delivery; notably, we consider peptide self-assembled nanocarriers from a broader “systems” perspective, at which the nanocarriers comprise peptides (including peptide conjugates/hybrids) which can be coassembled with drugs, metal ions, and/or peptides conjugated with drugs.

It is worth noting that small changes in the side chain can result in different supramolecular nanostructures. Numerous studies have demonstrated that diphenylalanine and its analogs form well-ordered nanotubes on their own.^23−25^ It was demonstrated that diphenylglycine could form spherical nanometric assemblies.^26−28^ In many respects, diphenylglycine is similar to diphenylalanine molecules. Under identical synthesis conditions, transmission electron microscopy (TEM) was employed to demonstrate that diphenylglycine peptides self-assemble into spherical nanometric structures, whereas diphenylalanine peptides self-assemble nanotubes.^26,27^ Diphenylglycine offers molecular properties similar to diphenylalanine, even though its structure is more rigid due to a lower degree of freedom associated with the absence of rotational freedom and higher steric hindrance.^26^ In the same context, a recent experimental and computational study on the double-fluorinated Fmoc-Phe derivatives, Fmoc-3,4F-Phe and Fmoc-3,5F-Phe, demonstrated the unique effects associated with self-assembly due to modifying the position of a single fluorine.^29^ The aforementioned studies demonstrate, among others, that small modifications in the peptide may result in changes in the supramolecular assemblies formed and highlight the importance of understanding self-assembly, in terms of studying the structural and physicochemical properties of the systems at the atomic and molecular level.

## Classes and Example Cases of Self-Assembling Peptide Materials as Cancer Drug Nanocarriers

### Cancer Drug Nanocarriers Formed from Short Peptide Self-Assembly

There are several studies on the engineering of novel cancer drug delivery systems using self-assembling peptide materials. A significant effort has been placed on short peptides, incorporating single-amino acid, dipeptide, and tripeptide systems, with or without modifications at the termini or amino acids. Using short peptides to engineer cancer drug nanocarriers is of great importance and is associated with minimalism, which is discussed in more detail in a subsequent section. Additionally, the importance of metal coordination is discussed in more detail in a subsequent section too. Importantly, we would like to highlight that several studies were published demonstrating short peptide self-assembled materials with the capacity to encapsulate different drugs, such as doxorubicin and 5-fluorouracil (ref (19) and references therein). While the scope of this paper is not to review such studies, we briefly highlight examples of single and dipeptide self-assembled peptide materials encapsulating particular cancer drugs. As for single peptide self-assembly, Singh et al. developed self-assemblies of derivatives of alanine peptides (including the addition of carboxamide and hydrazide at the carboxylic end), resulting in injectable hydrogels encapsulating doxorubicin, and showed that gels, injected at the site of tumors, could regress tumor load at the palpable stage.^30^ Additionally, Dube et al. showed that nanoparticles formed by Fmoc–Trp(Boc)–OH loaded with doxorubicin are more efficient in killing glioma cells compared to drugs alone; this suggested that such simplistic systems may be suitable for future applications in the field of drug delivery.^31^ As for dipeptides, Sun et al. demonstrated the formation of self-supporting hydrogels via the self-assembly of 5-fluorouracil dilysine conjugates, which exhibited promising in vitro cytotoxicity against different human tumor cell lines.^32^ A portion of the aforementioned studies and additional ones, some of which are presented in more detail in what follows, are summarized in Table 1.

**Table 1:** Self-Assembled Short Peptide-Based Cancer Drug Nanocarriers

short peptide^a^	self-assembled structures	drug	ref
Fmoc–Trp(Boc)–OH^b^	Nanoparticles	Doxorubicin	(31)
5-Fluorouracil dilysine	Hydrogels	5-Fluorouracil	(32)
Cyclo histidine–histidine–Zn(II)	Nanoparticles	Epirubicin	(33)
Arginine-α,β-dehydrophenylalanine	Nanoparticles	Doxorubicin	(34)
Tryptophan–phenylalanine–Zn(II)	Nanoparticles	Doxorubicin	(35)
Lysine–phenylalanine–glycine	Nanospheres and nanotubes	Doxorubicin	(36)
Boc-triphenylalanine–COOH	Hydrogel nanoparticles	Doxorubicin	(37)
d-Leucine–phenylalanine–phenylalanine	Supramolecular nanoparticles	5-Fluorouracil	(38)

a Peptide names are provided based on the nomenclature used in the corresponding studies.

b Fmoc-Trp(Boc)–OH: *N*-alpha-(9 fluorenylmethyloxycarbonyl)-*N*(in)-*tert*-butyloxycarbonyl-l-tryptophan.

In addition to the studies mentioned above related to FDA approved drugs, it is worth noting that other studies have focused on anticancer agents such as curcumin, a portion of which is reviewed in refs (19 and 39). Curcuminoids have been approved as “Generally Recognized as Safe”.^40^ We highlight one study according to which tetrapeptide Boc-Trp-Leu-Trp-Leu-OMe self-assembles into discrete nanospheres at a low concentration, while at higher concentrations, the nanospheres begin to cluster. Apart from serving like a conventional hollow sphere-based drug delivery vehicle entrapping curcumin and being capable to provide stimuli-responsive release, according to the authors, the particular prototype had the capacity to interact, stabilize, and intercalate hydrophobic dye carboxyfluorescein as well as curcumin even on the surface through aromatic interactions. The authors proposed that the dual curcumin encapsulation and intercalation capabilities suggest a prototype which can serve as a prospective drug delivery vehicle.^41^

### Cancer Drug Nanocarriers Formed from Conjugate/Hybrid or Larger Peptide Self-Assembly

In this section, we highlight studies on larger peptide self-assemblies and hybrid assemblies, which can serve as cancer drug delivery systems; larger peptides can be considered to comprise four or more amino acids. One particular example of copolymer self-assembly, which includes doxorubicin encapsulation, was performed by Qiao et al., who utilized Michael-type addition to synthesize a conjugate a cytotoxic peptide to poly(β-amino ester)s. The copolymers self-assembled into micelle-like, pH sensitive, nanoparticles. The authors demonstrated the ability of the micelles to encapsulate doxorubicin and additionally depicted that the drug loaded micelles could inhibit tumor growth effectively with an injection which was an order of magnitude lower than the corresponding amount of doxorubicin; the authors suggested that this is due to specific accumulation in tumor sites, in addition to efficient cellular entry as well as drug intracellular release.^42^ An additional example case comprises the study of Lu et al., who investigated and demonstrated methods on the characterization of the molecular states of cancer drug ellipticine encapsulated via EAK16-II, a self-assembling peptide.^43^ Furthermore, we would like to highlight an example case according to which a redox-responsive mesoporous silica nanoparticle was developed by Xiao et al. as a nanocarrier via noncovalent functionalization of mesoporous silica nanoparticles with amphiphilic peptides incorporating RGD. The authors suggested that the drug delivery self-assembled systems formed could comprise a facile but effective strategy toward smart cancer drug delivery.^44^

While this Perspective focuses on peptide self-assembling cancer drug nanocarriers, we consider it important to mention that self-assembling peptide systems alone have also been examined for their capacity to directly kill cancer cells (reviewed in ref (18)). Feng et al. examined “enzyme-instructed self-assembly” precursor analogues comprising N-capped D-tetrapeptide, a phosphotyrosine residue, and a diester or a diamide group. The authors demonstrated that the self-assembling capacities match the precursors’ anticancer activities.^45^ Additional mechanistic studies by Feng et al. demonstrated that the peptide derivative assemblies result in cell death.^45^ This work is highlighted also due to the demonstration of the correlation between thermodynamic properties of small molecules, such as self-assembling capacity, to the molecule’s anticancer efficacy against cancer cells.^45^ Additional studies associated with cancer drug nanocarriers formed from conjugate/hybrid or larger peptide self-assembly are provided in Table 2.

**Table 2:** Self-Assembled Conjugate/Hybrid or Larger Peptide-Based Cancer Drug Nanocarriers

conjugate/hybrid or larger peptide^a^	self-assembled structures	drug	ref
Phenylalanine-based pyrene conjugate	Hydrogels	Doxorubicin (and Vitamin B12)	(46)
Diphenylalanine peptides conjugated to folic acid/magnetic nanoparticles	Nanotubes	5-Fluorouracil	(47)
iRGD–lipid–polymer hybrid^b^	Lipid–polymer hybrid nanoparticles	Doxorubicin with sorafenib	(48)
Arginine–glycine–aspartic acid–polyethylene glycol–polylactide conjugate	Spherical micelles	Combretastatin A4	(49)
Cyclic arginine–glycine–aspartic acid–liposome conjugate	Liposomes	Doxorubicin	(50)
Arginine–glycine–aspartic acid peptide conjugated liposome	Liposomes	Cisplatin	(51)
Azabicycloalkane- and aminoproline-based cyclic arginine–glycine–aspartic acid semipeptide ligand	Liposomes	Doxorubicin	(52)
Octreotide	Nanoparticles	Doxorubicin	(53)

a Peptide names are provided based on the nomenclature used in the corresponding studies.

b iRGD corresponds to a nine amino acid cyclic peptide (sequence: CRGDKGPDC)).

### Computational Studies on Peptide Self-Assembled Cancer Drug Nanocarriers

In this section, we highlight a portion of computational studies on peptides, as well as amphiphilic-based self-assembled materials for cancer drug delivery. With the advancement of computing capabilities and computational methods, computational studies have been applied using a diverse set of tools and force fields to examine as well as facilitate the design of such materials. Using dissipative particle dynamics simulations, Guo et al. studied the microstructures of doxorubicin loaded/blank micelles self-assembled from cholesterol conjugated His10Arg10 at varying pH conditions.^54^ The authors showed that doxorubicin could be encapsulated efficiently in the micelles core and observed that, at pH higher than 6.0, the micelles possess stronger doxorubicin loading capacity owing to the histidine residues’ hydrophobicity, in comparison to pH values below 6.0.^54^ Thus, this transformation in the structure could facilitate doxorubicin release from the micelle core.^54^ The authors concluded that their results were qualitatively consistent with the experimental results, demonstrating that such computational methods could provide a useful tool in understanding and designing drug delivery systems.^54^ In a subsequent study, Guo et al. used a combination of atomistic and mesoscale simulations to investigate the interactions between each component of the drug delivery systems and mesostructures, respectively, and showed that this multiscale approach was successful and produced results in agreement with experiments, suggesting that such approaches are powerful tools to designing and developing drug delivery systems.^55^

Using coarse-grained force fields and molecular dynamics simulations, Kang et al., computationally investigated peptide–drug conjugates containing an aromatic cancer drug camptothecin. Their studies indicated that the self-assembly of a peptide–drug conjugate led to the formation of chiral filaments and suggested that the filament’s chirality is mediated via π–π stacking between drugs. The simulations performed allowed the identification of the self-assembly process according to which π–π stacking between drugs governs the self-assembly early, while a hydrogen bonding network is initiated later, contributing to the filament’s morphology. The authors suggested that their studies could provide valuable principles to rationally design supramolecular assemblies comprising peptide conjugates with aromatic segments.^56^ In two subsequent studies, the authors provided advanced insights into coarse-grained models, which were found to successfully recapitulate the growth of the molecular clusters, their interfacial structure and filaments helicity, the water dynamics within peptide–drug nanotubes, and their disassembly process.^57,58^

Moreover, Ashwanikumar et al., exploited RADA peptide self-assembly and showed that RADA-F6 peptide can be effectively utilized as a drug delivery system for the sustained release of 5-fluorouracil at basic pH. In this study, MD simulations were employed to elucidate how different pH conditions (e.g, acidic, neutral, and basic) have an effect on the peptides’ conformation and to uncover the mechanism of drug release, which was investigated in tandem with experiments.^59^ Finally, we would like to highlight a rather recent study according to which computations were used to identify cancer drugs which can serve as optimum solutions for spherical nanoassemblies formed by rhein–diphenylalanine peptide.^60^ Particularly, a structure-based virtual screening of small molecules was performed to select the suitable compounds with the capacity to be effectively delivered by the specific nanocarrier.^60^ Then, the authors sorted by binding energy and identified 15 superior and five inferior molecules; this prompted the authors to study and predict the coassembly ability of molecules using dissipative particle dynamics simulation.^60^ Interestingly, in line with computational results, the experiments depicted that camptothecin-encapsulated nanoassemblies have noteworthy advantages in particle size distribution as well as on the recrystallization-inhibitory effect, in comparison with norcantharidin.^60^

## Perspectives into Metal Coordination, Cyclization, and Minimalism

### Metal Coordination and Enhanced Fluorescence

A variety of analytical techniques have been employed to monitor the drug release kinetics, such as fluorescence detection,^61−63^ magnetic resonance imaging,^64^ ultrasound imaging,^65^ and electrochemistry measurement.^66,67^ Fluorescence has been considered as a fundamental and convenient tool in the quantification of the amount of drugs released in a complex intracellular environment.^68^ Fluorescence is produced via the radiative transition of excitation energy after light absorption. Fluorescence imaging can reflect timely the pharmacokinetics and biodistribution of drug delivery systems owing to its highly sensitive, noninvasive, and real-time as well as radiation-free features.^69−72^ Fluorescence is of critical importance to monitor drug release in vitro and in vivo, providing the capacity to accurately locate diseased tissues, avoid inappropriate drug dosage, and improve therapeutic efficiency.^72^

The discovery of green fluorescent protein has provided impetus for the design and engineering of novel fluorescent protein variants with improved photophysical and photochemical properties.^73−75^ One feature added to such engineered proteins is the capacity to coordinate with metal ions,^75−77^ and the engineering of such metal binding sites can alter the fluorescence, either by increasing or decreasing it.^75^ For example, metal-induced fluorescence alterations can be utilized to indicate the occurrence of particular metals within a solution or cell; consequently, a series of fluorescent proteins have been created to act as genetically encoded biosensors.^75^ In general, the metal-induced alteration in fluorescence can result due to static quenching^75,78^ or energy transfer between a colored metal ion and the chromophore^75,77^ or via perturbations associated with the protein’s structure.^75,76^

A series of fluorescent proteins were optimized for their binding properties to transition metal ions; such proteins can respond to metals with significant alterations in fluorescence intensity.^75^ Additionally, such proteins can serve as metal biosensors or imaging probes with fluorescence that can be modulated by metals. Such proteins can also act as metal biosensors or imaging probes whose fluorescence can be tuned by four different metals (Cu(II), Ni(II), Co(II), and Zn(II)).^75^ Transition metal ions nickel, copper, and zinc are vital in a series of pathophysiological and physiological pathways, and such fluorescent engineered proteins may serve as “sensitive transition metal ion-responsive genetically encoded probes that span the visible spectrum”.^75^ In general, during the last two decades, materials based on transition metal complexes were advantageously utilized in the design and engineering of fluorescence-responsive compounds for a series of applications, such as bioimaging and analytical probes, as well as lighting and switch devices. Emphasis is given on the abundant, less expensive, and environmentally “green” Zn(II) metal cation. Owing to the advantage of a wide variety of coordination geometries, in addition to elaborate molecular architectures, Zn(II) complexes provide versatility of the luminescent levels, in the solid state and in the solution state (reviewed in ref (79)).

Zn(II) complexes exhibit fluorescence tuning in intensity and/or emission maximum.^79^ Particularly, upon coordination, a fluorescence enhancement (chelation enhanced fluorescence) mechanism^80,81^ or fluorescence reduction (metal-binding-induced fluorescence quenching^82^) can occur.^79^ In addition, a qualitative fluorescence tuning can be observed due to the lowering of the excited state of the bonded ligand upon coordination.^79,81^ The fluorescence enhancement effect can often be attributed to the stabilization of the excited state in poorly emissive ligands upon coordination,^83^ and the chelation enhanced fluorescence effect can often be caused by Zn(II).^79^ Fluorescence quenching is not commonly observed in zinc complexes.^79^

Among the key interactions formed by Zn(II) with biological molecules, including but not limited to self-assembling peptides, is its interaction with histidine imidazole rings. Interactions between histidine and metal species are important in many biological processes.^84−87^ Such interactions have been studied extensively experimentally and theoretically. Zhou et al. reported that at acidic pH no direct interaction can be formed between Zn(II) and biprotonated histidine.^87^ At pH 7.5, one Zn(II) can be hexacoordinated with two histidines, while as pH increases in the range of 11 to 14, both the ND1 and NE2 sites can be deprotonated and serve as acceptors to bind either Zn(II) or water.^87^ The mechanism histidine to Zn(II) coordination was recently exploited by several groups for the development of materials encompassing such properties.^88−90^ In a more recent study by Song et al., first-principles calculations in conjunction with solubility experiments supported that the strong cation−π interaction formed between histidine and Zn(II) significantly affects histidine’s water affinity.^91^

### Peptide Cyclization and “Locking” of Particular Conformations

Cyclic peptides have been gaining attention as an alternative scaffold to noncyclic peptides.^92−94^ They are considered promising to serve as drug delivery systems due to several factors: (i) Cyclization imposes structural constraints, which could help in resisting proteases’ associated degradation in the blood, thereby augmenting their serum stability.^95^ (ii) Cyclization could enable passage via the cell membrane; this broadens the likely use of such peptides beyond extracellular targets.^96^ (iii) Cyclization in combination with assembly could result in the formation of particular conformations,^97^ and this can be a key to enhance the structural stabilization of assemblies into desired states. When compared against other peptide self-assembled nanostructures, cyclic nanostructures can possess advantageous properties, e.g., precise diameter controls; these could be modulated via the peptide sequences and lengths.^13^

### Minimalism and Importance of Reductionism

Despite the importance of larger cyclic peptides as drug nanocarriers, these are often complex to synthesize, and their production may be rather costly and require special settings. Consequently, there is a scientific and industrial need for minimalism in the development of mimetic, functional cyclic self-assembling peptide materials as drug nanocarriers with as much as possible simpler building blocks. A reductionist approach allowed the identification of extremely short peptide sequences, with diphenylalanine being presumably the most studied one. Particular peptide assemblies show notable mechanical, electrical, and optical characteristics, which were utilized for numerous applications in technology and biomedicine.^98^ The formation of nanostructures by short peptides, such as dipeptides, with remarkable properties, evidently establishes the importance of reductionism both in the design and synthesis of self-assembling peptides.^98^

### Example Studies Combining the Concepts of Metal Coordination, Cyclization, and Minimalism

Fan et al. demonstrated that nanoparticles formed by the self-assembly of tryptophan–phenylalanine can shift the intrinsic fluorescence signal from ultraviolet to visible. Importantly, the authors commented that the signal from visible emission allowed the nanoparticles to serve as imaging as well as sensing probes.^35^ The design’s inspiration comprised the mechanism resulting in the red shift observed in the yellow fluorescent protein as well as the enhanced fluorescence intensity observed in the green fluorescent mutant protein, BFPms1. The particular red shift in the yellow fluorescent protein is an outcome of π–π stacking as well as the enhanced intensity within BFPms1 due to the structure rigidification associated with Zn(II) binding.^35^ The data presented in this study depicted that the dipeptide nanoparticles are biocompatible and photostable and possess visible fluorescence properties as well as a narrow emission bandwidth.^35^ Furthermore, the dipeptide nanoparticles functionalized with doxorubicin and the MUC1 aptamer are capable of targeting cancer cells as well as provide the ability for imaging and monitoring drug release in real time. The aforementioned study provides a paradigm of self-assembling peptide cancer drug nanocarriers combining minimalism and metal coordination for enhanced fluorescence.^35^ Additionally, Sivagnanam et al. developed dipeptide-based self-assembled fluorescent nanoparticles representing a platform toward developing probes for cellular imaging, as well as systems for targeted drug delivery. In particular, they generated dipeptide-based nanoparticles from the self-assembly of tyrosine–tryptophan with Boc-protection, combined with structural rigidification provided by Zn(II);^99^ as a result, intrinsic fluorescent properties shifted from ultraviolet to visible. The nanoparticles formed encapsulated doxorubicin and facilitated intracellular drug delivery to kill cancer cells.^99^ This class of dipeptide-based nanoparticles proved to be photostable and biocompatible and have visible fluorescence signals that can be monitored in real-time during their cellular entry. The authors suggested that this approach could be used to deliver doxorubicin to cancer cells in vivo while sparing myocardium.^99^

**Figure 2:**
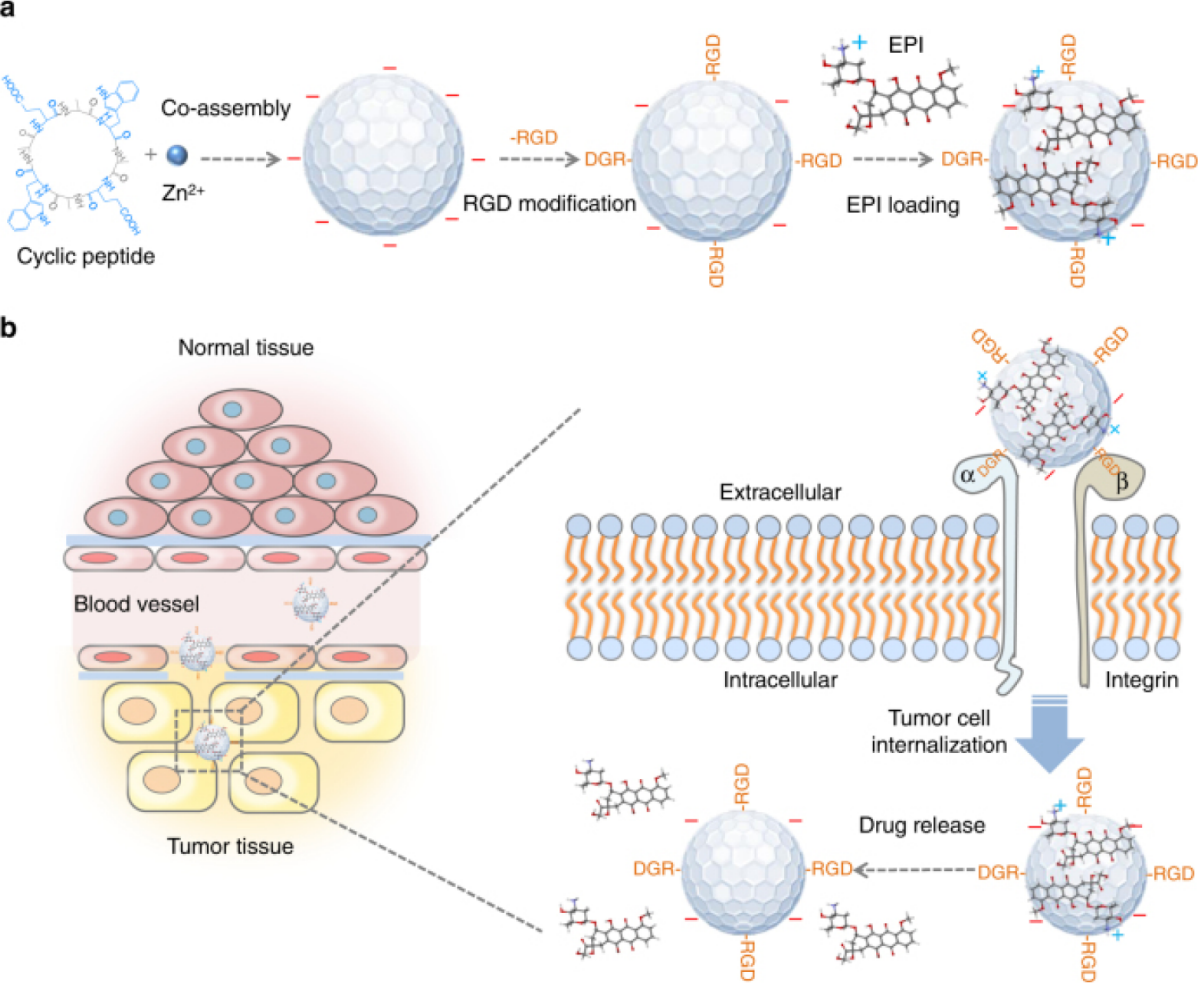
Schematic illustration of the synthesis of RGD-fluorescent nanoparticles with epirubicin and the targeted delivery of the drug into the EC cells. (a) The nanoparticles were produced via coassembly of Zn(II) and the cyclic peptides, and they were subsequently modified via RGD peptide moieties onto the surface of the nanoparticles. The drug loading was achieved via π–π stacking and electrostatic interactions. (b) The drug-loaded nanoparticles were used for the targeted imaging and deconstruction of EC cells as a result of their ability to actively target and possess enhanced penetration. Reproduced with permission from ref (100). Copyright 2018 Nature Publishing Group.

In addition to the study outlined above, Fan et al. designed novel fluorescent nanoparticles assembled by cyclic octapeptides to combine imaging and drug delivering for esophageal cancer.^100^ The nanoparticles were conjugated with RGD moieties to achieve tumor targeting and selectively target esophageal cancer cells via αvβ3 integrin; following that, the nanoparticles were embedded with epirubicin (Figure 2a).^100^ As shown in Figure 2b, arginine–glycine–aspartic acid fluorescent nanoparticles embedded in epirubicin nanoconjugates tend to accumulate in tumor tissues at a significantly higher rate than in normal tissues due to enhanced permeability and retention properties of the peptide (Figure 2b). The authors were able to monitor the drug delivery to tumor sites as well as therapeutic responses via near-infrared fluorescence through the self-assembling peptide–drug nanocarriers.^100^ The aforementioned study provides a paradigm of self-assembling peptide cancer drug nanocarriers combining cyclic peptide assembly and metal coordination for enhanced fluorescence.

Recently, our groups used a self-assembly strategy combining minimalism, cyclic-peptide self-assembly, and enhanced fluorescence driven by Zn(II) coordination. In summary, this strategy comprised cyclic l-histidine–d-histidine (Cyclo-HH) peptides, combining “self-encapsulation” of epirubicin and “self-locking” of Zn(II), resulting in high fluorescence efficiency of the resulting supramolecular peptide assemblies.^33,101^ Cyclo-HH was identified by a systematic, reductionist approach, similarly to diphenylalanine in the past, in the effort to identify the most stable, fundamental recognition unit that could form ordered structures with metal ion properties and encapsulate cancer drugs. Within the studies, we investigated the self-assembly properties of Cyclo-HH, epirubicin, and Zn(NO_3_)_2_ in isopropanol using a combination of simulations and experiments.^33^

**Figure 3:**
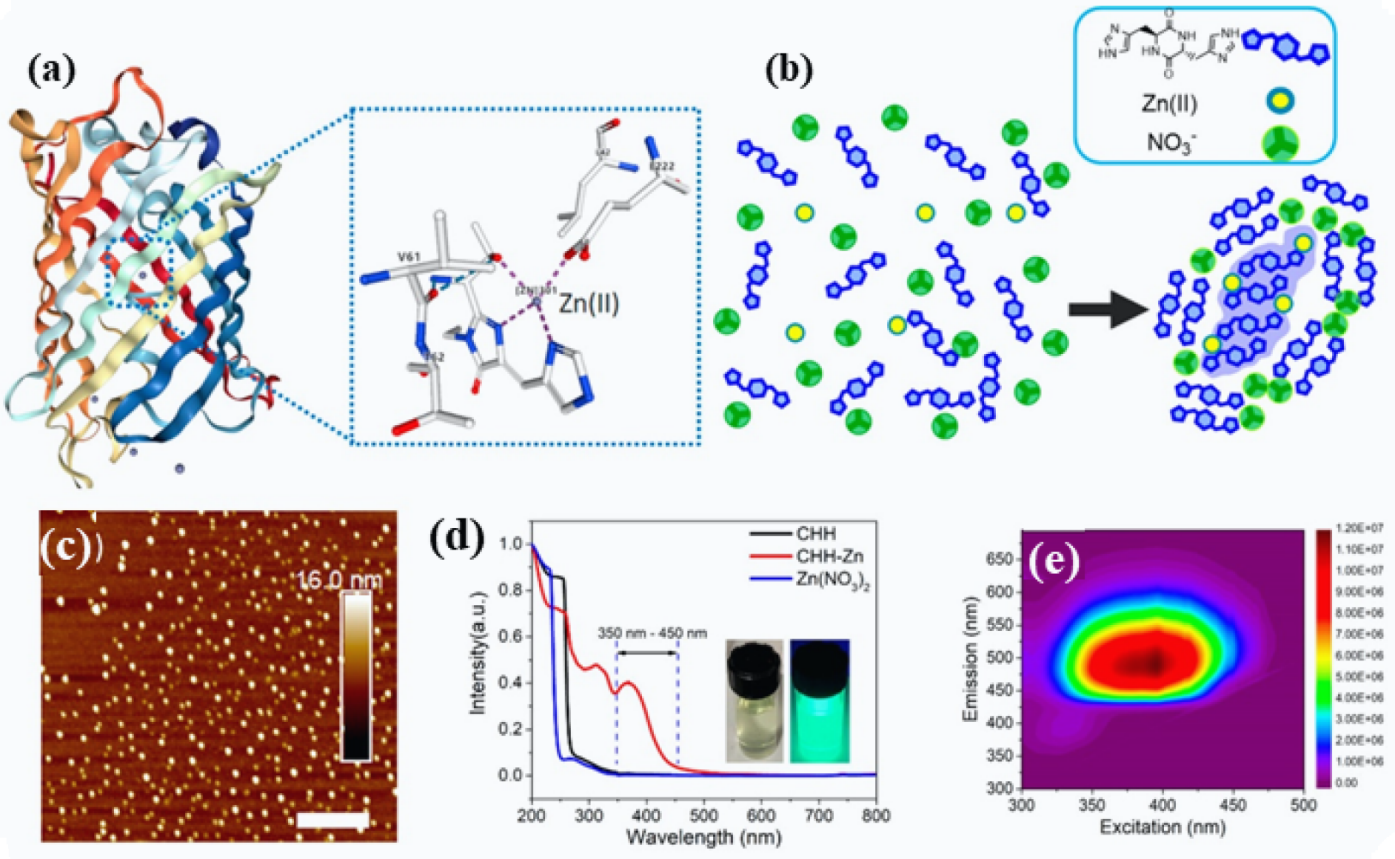
Design outline of the fluorescent self-assembled Cyclo-HH dipeptide. (a) The structure of BFPms1 and its coordination geometry with Zn(II). (b) An illustration of the computationally depicted self-assembly mechanism of Cyclo-HH, Zn (II), and NO_3_. (c) AFM micrograph shows ∼30 nm nanoparticles. Scale bar = 400 nm. (d) Normalized UV–Vis graph of Cyclo-HH-Zn, Cyclo-HH, and Zn(NO_3_)_2_ (Inset: Cyclo-HH-Zn under daylight (left) and UV lamp (right)). (e) Excitation–emission contour profiles of Cyclo-HH-Zn. Reproduced from ref (33). Copyright 2020 American Chemical Society.

Cyclo-HH along with other cyclic aromatic dipeptides were investigated in the past; according to the authors, the optical properties and morphologies of the cyclic dipeptides can be tuned, potentially leading to candidates aimed at supramolecular quantum confined materials comprising biocompatible alternatives with a series of biomedical and optoelectronic applications.^41^ Inspired by BFPms1 binding to Zn(II), we rationally designed a self-assembled peptide material, under controlled experimental conditions, by mixing Cyclo-HH peptide and Zn(NO_3_)_2_ to create nanoscale self-assembling peptide biological materials (Figure 3a,b).^33^ TEM and atomic force microscopy (AFM) were employed to confirm the presence of nanoscale biological materials with an average diameter of 30 nm (Figure 3c).^33^ In Figure 3d, the normalized UV–Vis absorption as well as excitation–emission matrix contour profiles of the assemblies are presented. Excited at 390 nm, the peptide nanoscale crystals displayed bright fluorescence emission centered at 500 nm (Figure 3e).^33^

**Figure 4:**
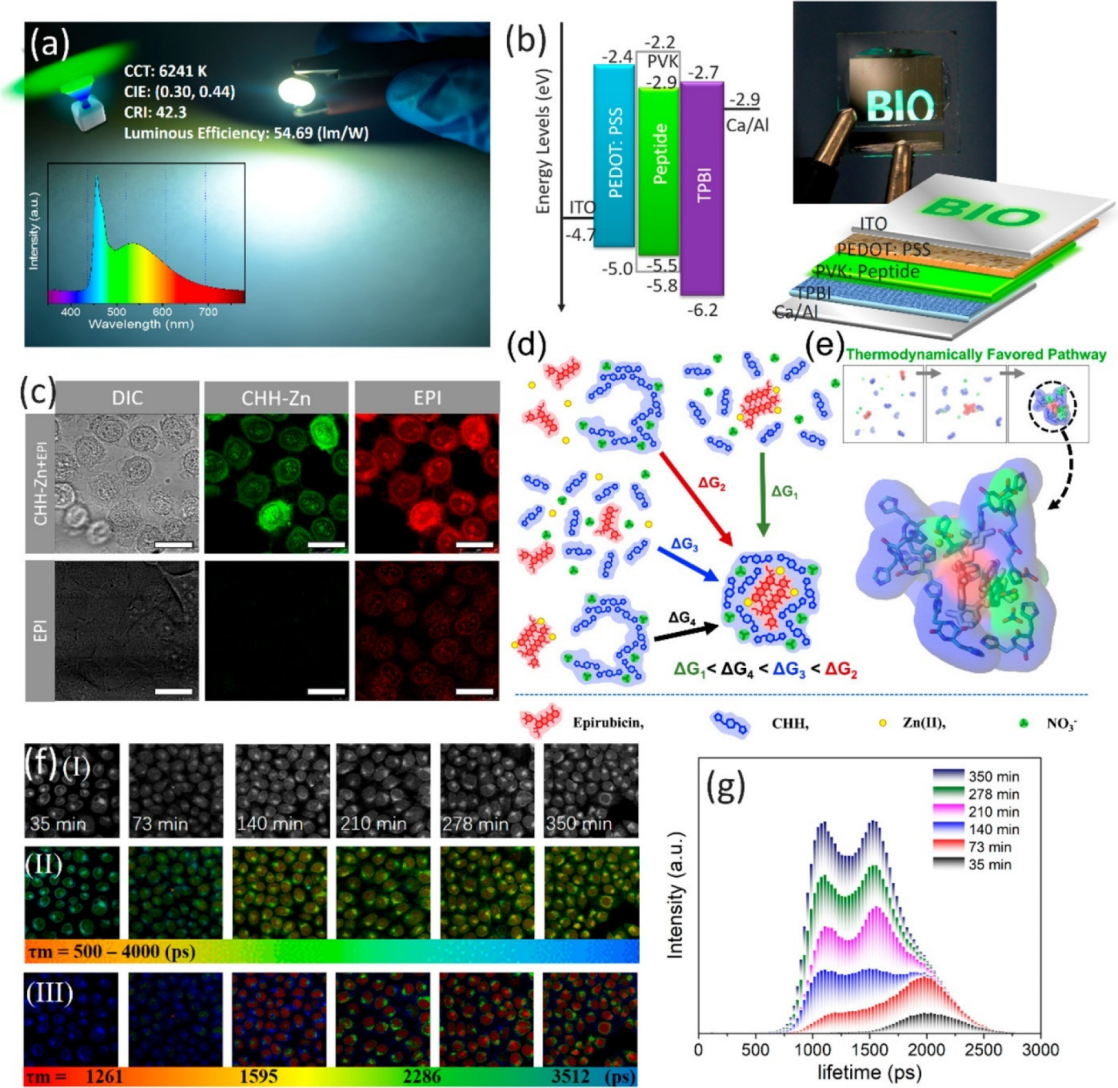
Cyclo-HH-Zn self-assembly applications. (a) Cyclo-HH-Zn application as a phosphor for green LEDs with a 54.69 lm/W luminous efficiency. (b) Structure, energy diagram, and photograph of the OLED in operation. (c) Confocal microscopy images of HeLa cells treated with Cyclo-HH-Zn+Epirubicin and Epirubicin alone. (d) Thermodynamic “thought” pathways of the coassembly of Cyclo-HH-Zn and Epirubicin. (e) Cyclo-HH-Zn encasing epirubicin has been observed in MD simulations. (f) The FLIM analysis of HeLa cells after being treated with Cyclo-HH-Zn+Epirubicin. (I) Bright-field, (II) FLIM images, and (III) phasor-separated and pseudocolored FLIM images of HeLa cells. (g) Epirubicin fluorescence lifetime histogram over time. Reproduced from ref (33). Copyright 2020 American Chemical Society.

Furthermore, their potential as emissive materials in the photo- and electroluminescent prototypes were studied (Figure 4a). Moreover, an OLED prototype was fabricated though the use of Cyclo-HH–Zn-blended PVK as an emissive layer (Figure 4b). Additionally, Cyclo-HH-Zn self-assembling biological materials were examined experimentally and found to be highly promising anticancer drug carriers.^33^

In order to investigate the drug delivery capacity of the biological self-assembly materials, HeLa cells were incubated with Cyclo-HH-Zn(II) or epirubicin alone. Cyclo-HH-Zn(II)-epirubicin increased the fluorescence intensity of intracellular epirubicin in cells significantly, demonstrating efficient uptake and release of epirubicin into the nucleus of HeLa cells from the Cyclo-HH-Zn(II) carrier (Figure 4c).^33^ By measuring the absorbance spectra of Cyclo-HH-Zn(II), Chen et al. confirmed that epirubicin loads into Cyclo-HH-Zn(II) peptide self-assembly by 15.67% and computationally studied the potential pathways of self-assembly (Figure 4d,e).^33^ The computational structural and energetic analysis provided a plausible mechanism for this formation: first, the individual or pairs of Cyclo-HH molecules pull Zn(II) from isopropanol into a more peptide-like environment,^33^ which was referred to as an “environment-switching” mechanism,^102^ and which was computationally depicted to enable the self-encapsulation of epirubicin primarily at the interior;^33^ this further facilitated the assembly of individual pieces of Cyclo-HH and NO_3_^–^ wrapping around, primarily at the exterior.^33^ As epirubicin is internalized into the cells, alterations in its fluorescence lifetime can reflect alterations in the subcellular microenvironment; these are indicative of drug release and transport. With longer incubation times (Figure 4f), more drug was released and, as a result, the fluorescence intensity of the drug gradually increased (Figure 4g), in combination with a decrease in the average lifetime.^33^ As early as 35 min after incubation, Cyclo-HH-Zn(II)-Epirubicin could bind to and accumulate around the cell membrane of HeLa cells and subsequently be released into the cytoplasm under an acidic environment and, following that, accumulate in the nucleus.^33^ The methodology of the aforementioned two studies^33,102^ was also presented in detail in a subsequent method-based paper.^103^

## Challenges in Nanomedicine Design Criteria

According to a recent review paper, the criteria associated with the design of anticancer nanomedicines so as to improve the anticancer efficacy as well as reduce toxicity can be summarized as follows: “(1) Nanomedicines increase drug accumulation through enhanced permeability and retention (EPR) in tumors to improve anticancer efficacy. (2) Long systemic circulation of nanomedicines with high plasma concentration reduces reticuloendothelial system clearance and decreases drug accumulation in the normal organs to reduce toxicity, and to enhance the EPR effect. (3) A universal nano delivery platform based on EPR and long systemic circulation can be developed to deliver different anticancer drugs”.^104^ Despite the fact that these criteria were confirmed in preclinical xenograft cancers, they are still under debate. Most nanomedicines for cancer showed failure in improving clinical efficacy.^104^ It was postulated that a “universal” nanodelivery platform that considers the same design-based criteria for various drugs is infeasible; it was also suggested that drug specific nanodelivery systems need to provide a solution for the intrinsic deficiencies of drugs, which are associated with the pharmacokinetic, pharmacodynamic, and physicochemical properties of the drugs in combination with the nanocarriers, with the aim to improve efficacy and safety.^104^ In a subsequent study on the reappraisal of anticancer nanomedicine design criteria, the data produced suggest that the design would need to be nanocarrier specific, drug specific, and cell type specific to augment success rates of nanomedicines in clinical testing.^105^ Overall, the study provided insights on why the design criteria considered in several preclinical models could lead to successful or poor clinical translation for clinical efficacy/safety in cancer patients.^105^

## Future Perspectives: Addressing a Portion of Challenges via Self-Assembling Peptide Systems

Carriers of the order of micro- and nanoscale, loaded with drugs with the capacity to target tumor sites, possess a significant potential to provide a solution for the challenges associated with the treatment of resistant or aggressive cancers. Effective targeting can be beneficial for a more favorable drug biodistribution and as a result higher tolerable doses as well as decreased systemic toxicity.^106^ When one takes into consideration that certain chemokine receptors are overexpressed in particular cancer cells, such as CCR4, CCR5, CCR7, and CXCR4,^107−109^ it is important to consider designing nanocarriers with chemokine receptor targeting properties, aiming to target cancer cells. The binding of chemokine-to-chemokine receptors has been the focus of many studies in the past. Among others, Qing et al. developed chimeric proteins via switching the N-terminus as well as three extracellular loops between different chemokine receptors to facilitate the elucidation of the native ligand interaction mechanism.^110^ Particularly, CCR5^QTY^ and CXCR4^QTY^ were redesigned to construct chimera A, which comprised a replacement of EC loops of CCR5^QTY^ with GS linkers of the same length, as well as chimera B, which comprised a replacement of N-terminus and EC loops of CCR5^QTY^ with the N-terminus and EC loops of CXCR4. According to the experimental results, chimera B displayed reduced affinity to CXCL12, which is a natural ligand of CXCR4, and significantly decreased affinity to CCL5, which is the natural ligand of CCR5. Chimera A displayed an 8-fold decrease in CCL5 affinity and no affinity for CXCL12.^111^ Interestingly, the results were in line with computationally predicted complex structures by Tamamis and Floudas comprising CCR5 in complex with CCL5, according to which the first 6 residues of the N-terminus of CCL5 are inserted into the CCR5 transmembrane region, while the 7–15 N terminus residues of CCL5 interact with CCR5 N-terminus and extracellular loops.^112^ According to a recent review by Qing et al., the experimental results agreed with the computational structural models by Tamamis and Floudas^113^ and enabled the illustration of the relative contributions from the N-terminus, extracellular loops and transmembrane regions for interactions in native receptors.^111^ The experimental results can provide impetus for constructing hybrid proteins, integrating and fine-tuning functions associated with multiple receptor templates with a rigid backbone structure.^111^ In addition, the agreement between the experiments and previous computations supports further the validity of the computational structural models^112,113^ and suggests that these models may possibly aid in the design of self-assembled cancer drug nanocarrier systems with cancer-targeting properties. Several studies explored the utilization of ligands binding to CXCR4 toward the improvement of drug delivery for tumors overexpressing CXCR4. Short CXCR4-binding peptides are among the most popular ligands.^106^

Self-assembly occurs through noncovalent interactions between the molecular building blocks and can also involve coassembly, when diverse components assemble in the system. The interactions include a diversity of nonpolar and polar interactions between the components, as well as between the components and the solution. It is important to note that the environment, which may include the solution, and other factors, including pH, temperature, etc., are key to the formation of the resulting nanostructures. The design of such nanostructures, particularly to serve as cancer drug nanocarriers, can be performed by intuition, guided by experimental structure resolution methods, as well as by computational methods such as simulations, which can be used to study and predict their molecular self-assembly properties.

Dr. Tamamis’ and Dr. Gazit’s vision is outlined as follows: Computational methods can, in one direction, potentially be used to understand and potentially “tune” the properties of currently known self-assembling peptide systems, guiding the addition, removal, or alternation of the components of the environment, in a “systems approach”, toward and ultimately leading to peptide systems, with drug specific and nanocarrier specific modulated properties. In this direction, the design criteria of the self-assembling peptide system may be constantly improved and optimized for each drug to enhance encapsulation. Furthermore, computational methods can, in another direction, also be used in the future to design drug specific and nanocarrier specific self-assembling systems, which do not necessarily rely on the use of known self-assembled systems but rather rely on the use and development of novel computational approaches (e.g., utilizing artificial intelligence, screening, etc.) to design such systems; in this direction as well, the design criteria can also be constantly improved and optimized as mentioned above. Therefore, computationally guided or computationally designed systems, which can also be modulated to allow cancer cell-targeting properties could be a promising future direction toward potentially addressing a portion of the challenges associated with cancer drug delivery agents. For example, by suitably exploiting the physics and chemistry principles of peptide–peptide interactions, novel self-assembling peptide systems with chemokine receptor targeting properties can potentially be designed as a promising future direction in the field of novel nanocarriers for cancer drugs; this can be considered a potential advantage of self-assembling peptide materials. Moreover, the cancer drug encapsulation that could be provided in such designed self-assembling peptide systems could increase circulation time and target specificity, due to the prevention of premature enzymatic, chemical, pH, or hydrolytic degradation.^14^ Nevertheless, one needs to consider the importance of the fact that the resulting nanostructure must destabilize and release the drug in the presence of the biological target for the drug to exert is pharmacological activity and thus, importantly, consider the mechanism through which the cargo will be released.^14^ Computational and experimental approaches can further be used to design the nanostructures to disassemble and release their cargo in the presence of an overexpressed enzyme or through a pH sensitive release, taking into consideration that normal tissue is reported to have a pH of approximately 7.4, while cancerous tissues have been reported to have a lower pH of 6.2 to 7.4.^14,114,115^

## Concluding Remarks

Cancer is a major health problem and a complex disease. Drug delivery into cancer cells is considered among the purposes of cancer therapy.^116^ Compared to conventional drugs, nanoparticle-based drug delivery can be considered advantageous, due to its improved stability and biocompatibility, enhanced permeability and retention effect, and precise targeting.^117^ Nanomaterial-toxicological issues need to be considered within the framework of novel improved cancer therapeutic strategies; additionally, combination therapeutic regimens for different cancer types needs to be addressed due to the diversity of mechanisms involved in cancer.^7^ Combination therapy with nanomaterial-based drug carriers needs further investigation at both preclinical and clinical levels.^7^ A series of aspects need to be taken into consideration, including localization, biodistribution, biocompatibility, and efficacy of nanodrug systems in vivo, in our effort to achieve precision cancer diagnosis and therapy.^7^ Nevertheless, despite their promise, few nanomaterial-based systems are in clinical trials.^14^

The design of novel self-assembling peptide materials for cancer drug encapsulation represents a promising additional direction in the field of cancer therapeutics. While self-assembled peptide nanocarriers for cancer drugs should not be considered a panacea to the challenges of cancer drug delivery, we consider that the intrinsic advantages of self-assembling peptide materials, along with the increasing progress in computational and experimental approaches for their study and design, could possibly lead to novel classes of systems that may provide additional alternatives to current approaches and/or provide a seed for novel multicomponent systems which may partly incorporate peptide self-assembled materials for cancer drug delivery. Importantly, due to the increasing advancement of computing capabilities and the development of novel computational study and design approaches in peptide self-assembly (e.g., refs (118−136)), computations can play a key role in designing multicomponent systems and/or optimizing existing systems, based on feedback that can be provided by experimental in vitro/in vivo studies. Dr. Tamamis and Dr. Gazit envision that the design of novel self-assembling peptide cancer drug nanocarriers can be significantly enabled through integrated and synergistic experimental and computational approaches, with computational feedback provided to experiments and vice versa, ultimately leading to optimized systems and continuous improvment based on the design criteria and ultimately updating and refining the design criteria; such integrative and synergistic approaches could possibly provide means to jointly combat many issues to be addressed in the field, so that novel and improved cancer drug delivery systems, such as self-assembling peptide materials, can potentially and ultimately become translatable from the lab to the clinic.
